# No Evidence for AID/MBD4-Coupled DNA Demethylation in Zebrafish Embryos

**DOI:** 10.1371/journal.pone.0114816

**Published:** 2014-12-23

**Authors:** Nobuyoshi Shimoda, Kentaro Hirose, Reiya Kaneto, Toshiaki Izawa, Hayato Yokoi, Naohiro Hashimoto, Yutaka Kikuchi

**Affiliations:** 1 Department of Regenerative Medicine, National Institute for Longevity Sciences, National Center for Geriatrics and Gerontology, 35 Gengo, Morioka, Obu, Aichi 474-8522, Japan; 2 Department of Biological Science, Graduate School of Science, Hiroshima University, Kagamiyama 1-3-1, Higashi-Hiroshima, Hiroshima 739-8526, Japan; 3 Max Planck Institute of Biochemistry, Am Klopferspitz 18, 82152 Martinsried, Germany; 4 Graduate School of Agricultural Science, Tohoku University, 1-1 Tsutsumi-Dori Amamiya-Machi, Aoba-Ku, Sendai 981-8555, Japan; Peking University Cancer Hospital and Institute, China

## Abstract

The mechanisms responsible for active DNA demethylation remain elusive in Metazoa. A previous study that utilized zebrafish embryos provided a potent mechanism for active demethylation in which three proteins, AID, MBD4, and GADD45 are involved. We recently found age-dependent DNA hypomethylation in zebrafish, and it prompted us to examine if AID and MBD4 could be involved in the phenomenon. Unexpectedly, however, we found that most of the findings in the previous study were not reproducible. First, the injection of a methylated DNA fragment into zebrafish eggs did not affect either the methylation of genomic DNA, injected methylated DNA itself, or several loci tested or the expression level of aid, which has been shown to play a role in demethylation. Second, aberrant methylation was not observed at certain CpG islands following the injection of antisense morpholino oligonucleotides against aid and mbd4. Furthermore, we demonstrated that zebrafish MBD4 cDNA lacked a coding region for the methyl-CpG binding domain, which was assumed to be necessary for guidance to target regions. Taken together, we concluded that there is currently no evidence to support the proposed roles of AID and MBD4 in active demethylation in zebrafish embryos.

## Introduction

We recently reported age-dependent decreases in 5-methylcytosine (5mC) in the zebrafish genome, and found that these were observed earlier than initially expected [1]. A slight decrease in global methylation was noted when late embryos (48 hours post fertilization; 48 hpf) were compared with early embryos (4 hpf, blastula stage), and we identified the CpG island shore as the preferred region for age-related hypomethylation by bisulfite sequencing [1]. Although the mechanisms responsible for hypomethylation remain unknown, a failure in the maintenance of a methylation pattern or the enzymatic removal of 5mC at CpG island shores during aging has been implicated.

Collas first reported that an *in vitro*-methylated plasmid injected into fertilized zebrafish eggs was gradually demethylated starting from 1∼2 hpf until 12 hpf, which indicated the presence of demethylation activity in zebrafish embryos [2]. Demethylation was found to be replication-independent, thereby suggesting an enzymatic reaction that converted 5mC into cytosine (C) in zebrafish embryos. Rai et al. also injected methylated DNA fragments (M-DNA) into zebrafish eggs and demonstrated that exogenous M-DNA elicited demethylation of not only M-DNA itself, but also the genomic DNA of M-DNA-injected zebrafish embryos [3]. Although the biological significance of demethylation elicited by this exogenous DNA was not described in their study, by utilizing this artificial demethylation system, Rai et al. tested for candidate enzymes, the function of which may explain the possible scenario for demethylation: conversion of 5mC to thymine (T) via deamination, followed by the removal of the mismatching T, and subsequent replacement with C through base excision repair [3]. The candidates in the deamination processes that were examined included all three members of the activation-induced deaminase (AID)/Apolipoprotein B RNA-editing catalytic component (APOBEC) deaminase family in zebrafish; AID, Apobec2a, and Apobec2b [4], [5]. Although zebrafish AID was recently shown to deaminate methylated cytidines *in vitro* [6], whether Apobec2a and Apobec2b exhibit deaminase activity has yet to be determined. In this context, it is important to note that mouse Apobec2 did not exhibit deaminase activity, and this is in contrast to its paralogs, Apobec1 and some Apobec3 genes, which have been shown to deaminate particular RNAs and DNAs, respectively [7], [8]. Another candidate enzyme tested was a likely ortholog of human G: T-mismatch glycosylase, methyl-binding domain protein 4 (MBD4), which removes T mispairing with G *in vitro* [9], [10]. MBD4 is also known to play a role *in vivo* by suppressing mutations at CpG sites in mammalian genomes [11], [12]; however, a defect in DNA methylation was not observed in *mbd4* knockout mice, which are viable and fertile [11]. The non-enzymatic factor Growth arrest and DNA-damage-inducible protein 45 alpha (Gadd45a), which was shown to be involved in the global DNA demethylation of human culture cells [13], was also examined, while contradictory results were obtained using the same cells or *Gadd45a* ^−/−^ mice [14], [15]. Rai et al. demonstrated that the knockdown of AID or MBD4 caused the aberrant methylation of CpG islands and that overexpression of a heterogeneous combination of genes, zebrafish *aid* and human *MBD4* elicited DNA demethylation in zebrafish embryos, which was promoted by the additional expression of *gadd45a* [3]. Based on these findings, together with other supporting data, they proposed a mechanism for demethylation that is exerted by the concerted action of the three products: the conversion of 5mC to T via deamination by AID, followed by thymine base excision repair by MBD4, can occur in zebrafish embryos, and is facilitated by Gadd45a, which may serve as a scaffold to physically and/or functionally couple AID and MBD4. To the best of our knowledge, this was the first evidence for the involvement of T-G mismatches in vertebrate DNA in global demethylation and provided the rationale for the three factors to be investigated for active demethylation in mammalian cells; however, questions were raised on their model [16]. Following this study, Rai et al. suggested that the proposed demethylation machinery may be involved in regulating intestinal cell fating [17].

We speculated whether the active demethylation system described by Rai et al. [3] in zebrafish embryos may be involved in the age-related hypomethylation of CpG island shores. To investigate this possibility further, we examined the reproducibility of several experiments in their previous study [3], and demonstrated that most of their findings were not reproducible. This finding, together with the absence of actively-demethylated loci in the genome of zebrafish embryos [18], [19], cast doubt on the occurrence of active demethylation during the early development of zebrafish.

## Results

### No discernible hypomethylation of genomic DNA or methylated-DNA (M-DNA) itself was observed following the injection of M-DNA into zebrafish fertilized eggs

Rai et al. reported that the injection of 200 pg of *in vitro*-methylated DNA (M-DNA) in fertilized zebrafish eggs caused not only the significant demethylation of M-DNA itself, but also demethylation of 20%–40% of the bulk genome, both of which peaked at 13 hpf, which corresponded to the early somite stage of zebrafish development [3]. This artificially-induced demethylation system was utilized throughout the study to identify candidate genes, such as *aid* and *mbd4*, in the demethylation activity observed in zebrafish embryos. Therefore, M-DNA-dependent demethylation represented a fundamental experiment to confirm the reproducibility of their experimental results.

**Figure 1:**
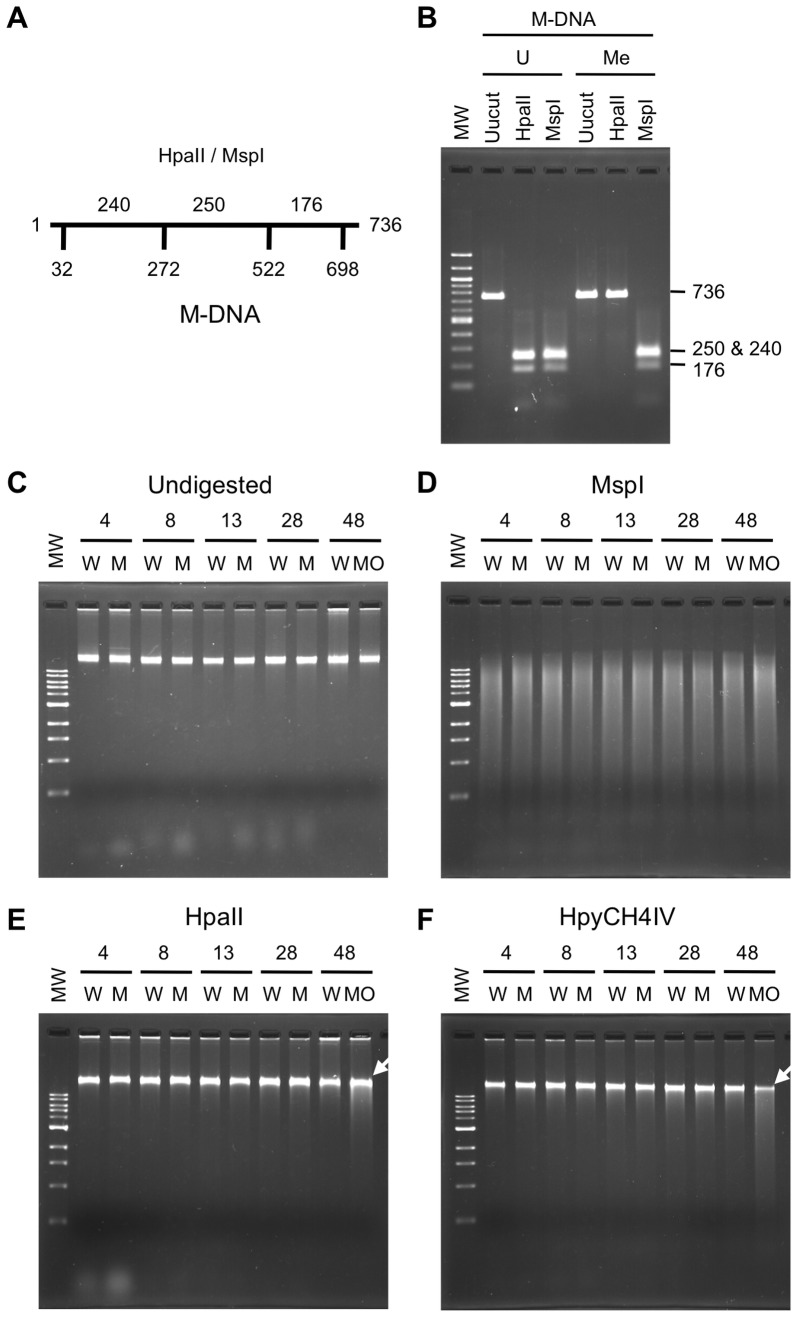
Absence of genome demethylation following the injection of a methylated DNA fragment into zebrafish eggs. (A) A schematic structure of the 736-bp DNA template used for *in vitro* methylation by HpaII methylase to generate M-DNA. Numbers below the horizontal line indicate the positions of HpaII/MspI sites subject to methylation. Numbers above the line show the lengths of the fragments generated when completely digested with HpaII or MspI. (B) Verification of *in vitro* methylation of the 736-bp DNA template. The unmethylated DNA fragment (U) was susceptible to HpaII, a methylation-sensitive restriction enzyme, as well as MspI, the methylation-insensitive isoschizomer of HpaII. Methylation of the fragment (Me) conferred resistance to DNA to the digestion by HpaII. (C) Undigested genomic DNA of control (W), M-DNA-injected (M), and *dnmt1* MO-injected (MO) embryos at the indicated time points (hours post fertilization; hpf) were run on an agarose gel. (D, E, F) The same genomic DNAs as those used and shown in (C) were digested with MspI (D), HpaII (E), or HpyCH4IV (F), and run on an agarose gel. The molecular weight markers of DNA loaded on the first lanes of gels, shown as MW, were 100 bp (B) or 1 kb ladders (C, D, E, and F). Note that smearing down of high-molecular DNA, thereby reducing the methylation level, was discernible only in the genomic DNA from *dnmt1* MO-injected embryos (white arrows in E and F).

To determine whether the demethylation system was reproducible, we prepared the same DNA fragment (736 bp) as that used by Rai et al. [3] (Fig. 1A), and methylated it *in vitro* using HpaII methylase (Fig. 1B). Methylation was confirmed at four CCGG sites in the fragment by its resistance to HpaII, which cleaved the tetranucleotide when C was not methylated (Fig. 1B). We injected 200 pg of M-DNA per fertilized zebrafish eggs as Rai et al. did. The intactness and quantity of genomic DNA from M-DNA-injected and -uninjected embryos were examined by running undigested genomic DNA on an agarose gel (Fig. 1C). The purity of genomic DNA was verified by digestion with MspI, which is a methylation-insensitive isoschizomer of HpaII (Fig. 1D). To prepare a reference of a globally demethylated genome, we suppressed the activity of DNA methyltransferase 1 (Dnmt1) using an injection of antisense morpholino oligonucleotides (MO) against the *dnmt1* gene [20], [21] into zebrafish eggs, and then extracted genome DNA at 48 hpf. The knockdown of *dnmt1* resulted in approximately 20% hypomethylation at the several loci examined by bisulfite sequencing [22], which should be similar to the moderately demethylated genome in M-DNA-injected embryos [3]. The digestion of the genome from *dnmt1* MO-injected embryos by HpaII or HpyCH4IV, another methylation-sensitive enzyme, was more sensitive to the methylation-sensitive enzymes than the control genome, which indicated that the methylation level in *dnmt1* MO-injected embryos was lower than that in the control embryos (white arrows in Fig. 1E and 1F). In contrast, we observed no significant differences in digestion patterns between the genomes from M-DNA-injected and control embryos at any of the developmental stages examined (Fig. 1E and 1F), even though a M-DNA injection was shown to cause genome-wide demethylation that was detectable with HpaII digestion, followed by electrophoresis on agarose gels [3].

**Figure 2:**
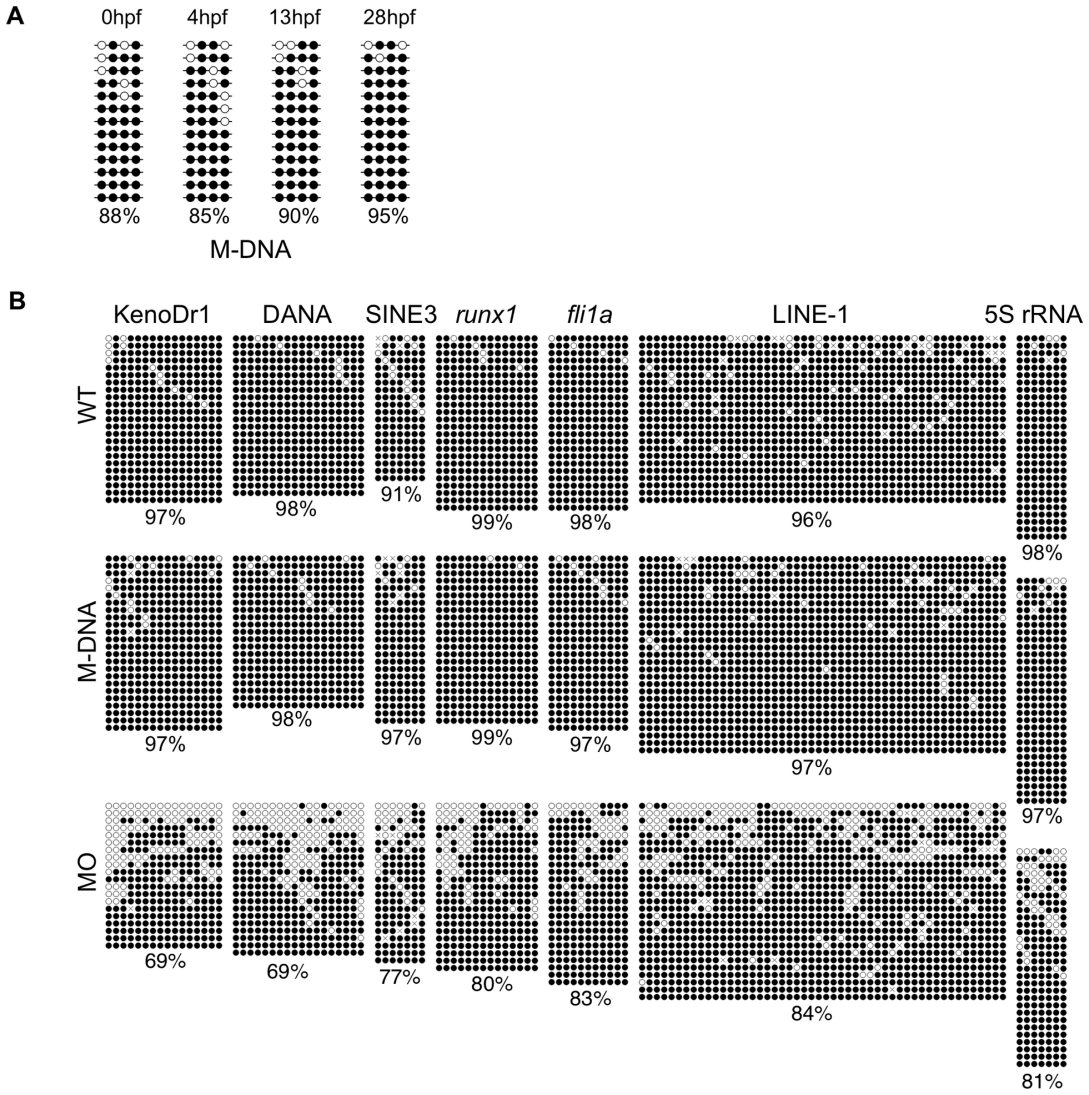
No demethylation at M-DNA or randomly chosen loci was detected by the M-DNA-injection into eggs. (A) Bisulfite sequencing revealed no significant changes in the methylation levels of injected M-DNA recovered from 10 embryos at any of the indicated time points. (B) Bisulfite sequencing analysis showed that the conserved regions of the five repetitive sequences (KenoDr1, DANA, SINE3-1a, LINE-1, and 5S rRNA) or confined regions of two single genes (*runx1* and *fli1a*) were highly methylated in M-DNA-injected embryos at 13 hpf (M-DNA) as in controls (WT), whereas moderate hypomethylation was observed in the equivalent regions in *dnmt1* MO-injected embryos (*dnmt1* MO). Black and white circles are methylated and unmethylated cytosines, respectively. Crosses denote the positions at which CpG was absent due to polymorphisms. Values represent the ratio of the numbers of methylated cytosines in the total number of CpG dinucleotides in the regions surveyed.

Rai et al. also demonstrated that M-DNA injected into embryos clearly lost its methylation at 13 hpf [3], which was in accordance with the findings reported by Collas [2]. To confirm this, we recovered injected M-DNA at 13 hpf from embryos and examined its methylation using bisulfite sequencing. In contrast to the findings of Collas [2] and Rai et al. [3], we demonstrated that injected M-DNA remained highly methylated at any time point, including 13 hpf (Fig. 2A). For these experiments we used the zebrafish line, named AB/Tü, which was derived from a cross of AB and Tübingen (Tü) lines. On the other hand, Rai et al used Tü line. To examine if the discrepancies we observed above could be attributed to the difference in zebrafish lines, we performed the same experiments with Tü line but were still unable to reproduce the results that Rai et al. described (S1 Fig.). In accordance with these results, we observed no demethylation in any of the randomly-chosen seven sequences, which included three types of repetitive sequences (two LINEs; KenoDr1 and LINE-1, two SINEs; DANA and SINE3-1a, and 5S rRNA) and two unique sequences (*runx1* and *fli1a*), in M-DNA-injected embryos at 13 hpf (Fig. 2B). On the other hand, as we previously reported [22], a reduction was observed in methylation levels in all of the selected sequences from *dnmt1* MO-injected embryos (Fig. 2B). Thus, we concluded that the injection of M-DNA into zebrafish eggs had no discernible effect on the methylation level of genomic DNA from M-DNA-injected zebrafish embryos or the injected M-DNA itself. These results demonstrated that the first and fundamental experiments of Rai et al. [3] were not reproducible.

### Injection of M-DNA into zebrafish eggs does not increase the expression level of *aid* and *apobec2a*

**Figure 3:**
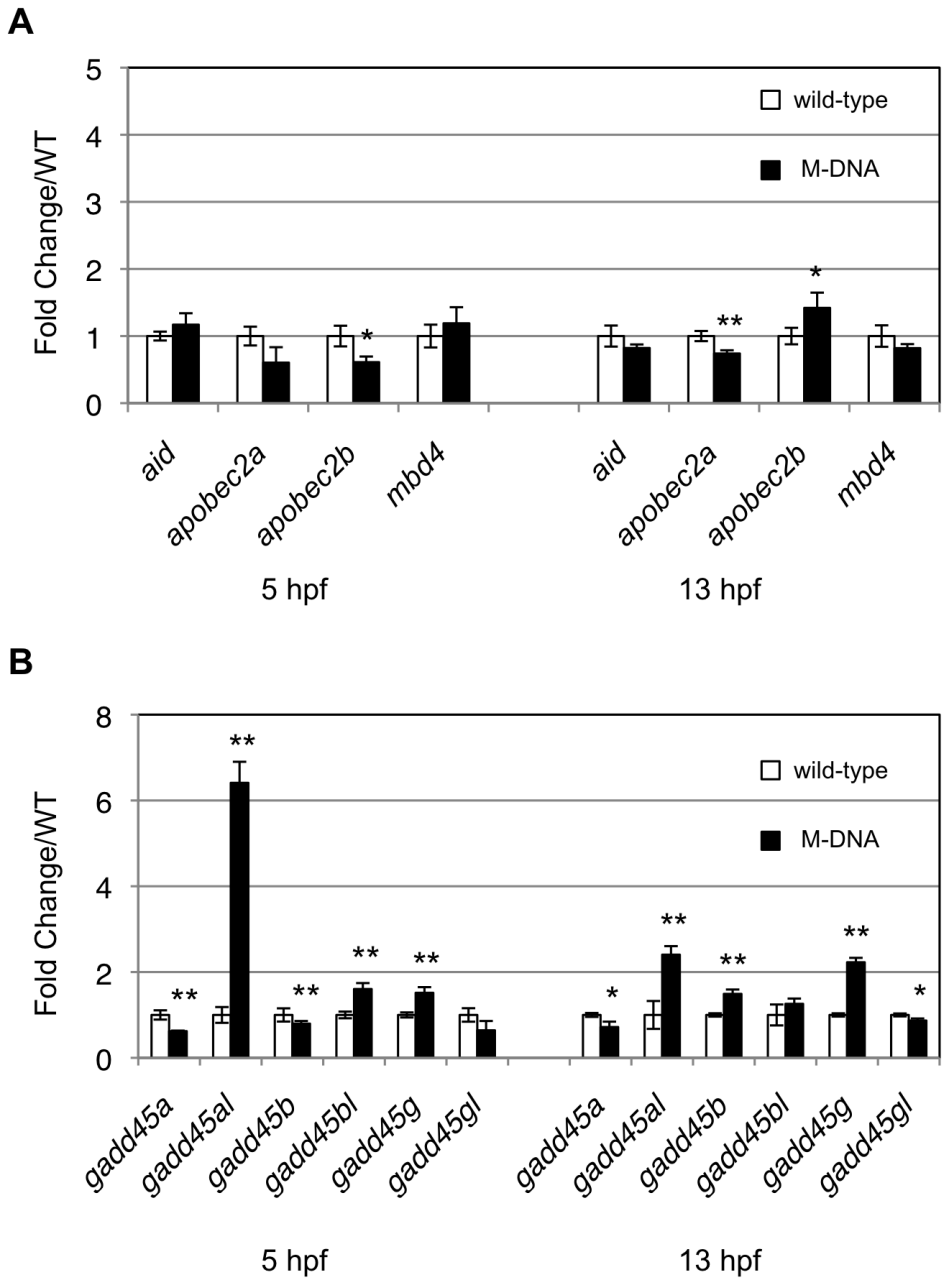
M-DNA did not induce the expression of *aid* or *apobec2a*. (A) The expression levels of AID/Apobec family genes at the indicated time points were quantified by qPCR, and these expression levels in wild-type embryos (white rectangles) were compared with those in M-DNA-injected embryos (black rectangles). (B) The expression levels of Gadd45-family genes between wild-type and M-DNA-injected embryos were compared using qPCR. Gene expression was normalized against *rpl13a* (*ribosomal protein L13a* gene) and data represent the mean ± standard error of the mean (s.e.m.) from three independent experiments; **P<0.01 and *P<0.05.

Rai et al. also reported that the injection of M-DNA into zebrafish eggs increased the expression levels of all three annotated members of the AID/Apobec deaminase family, i.e. AID, Apobec2a, and Apobec2b [3]. Based on this finding, these genes were selected as candidates that could play roles in artificially induced demethylation, as described above. However, we wanted to confirm whether the expression of these deaminase genes was upregulated in an M-DNA-dependent manner because the M-DNA injection did not cause demethylation in zebrafish embryos. We examined the expression of the deaminase genes by quantitative real time PCR (qPCR), and found no significant increases in the expression of either *aid* or *apobec2a* at 13 hpf (Fig. 3A). In contrast, although the expression of *apobec2b* was upregulated (Fig. 3A), the upregulation level of this gene (approximately 1.5-fold change/WT) was lower than that reported previously [3] (approximately 5-fold change/WT), and no upregulation of *apobec2b* was observed when Tü line was used (S2A Fig.). Our results indicated that *aid* would not be selected as a candidate gene that could be involved in artificially induced demethylation. In addition, Rai et al. showed that four of the six Gadd45 family genes examined, namely *gadd45a*, *gadd45a like* (*gadd45al*), *gadd45b*, and *gadd45g*, were all markedly upregulated either at 5 hpf, 13 hpf, or at both time points by the M-DNA injection, and *gadd*45*a* was chosen for further analyses and was shown to enhance demethylation [3]. We examined the expression of all six *gadd45* genes, and showed that none of them, except for *gadd45al,* were markedly upregulated by M-DNA (Fig. 3B and S2B Fig.). Rather than being increased, the expression level of *gadd*45*a* in M-DNA-injected embryos was slightly lower than that in control embryos at 13 hpf (Fig. 3B).

### Zebrafish MBD4 lacked a methyl-CpG binding domain (MBD)

**Figure 4:**
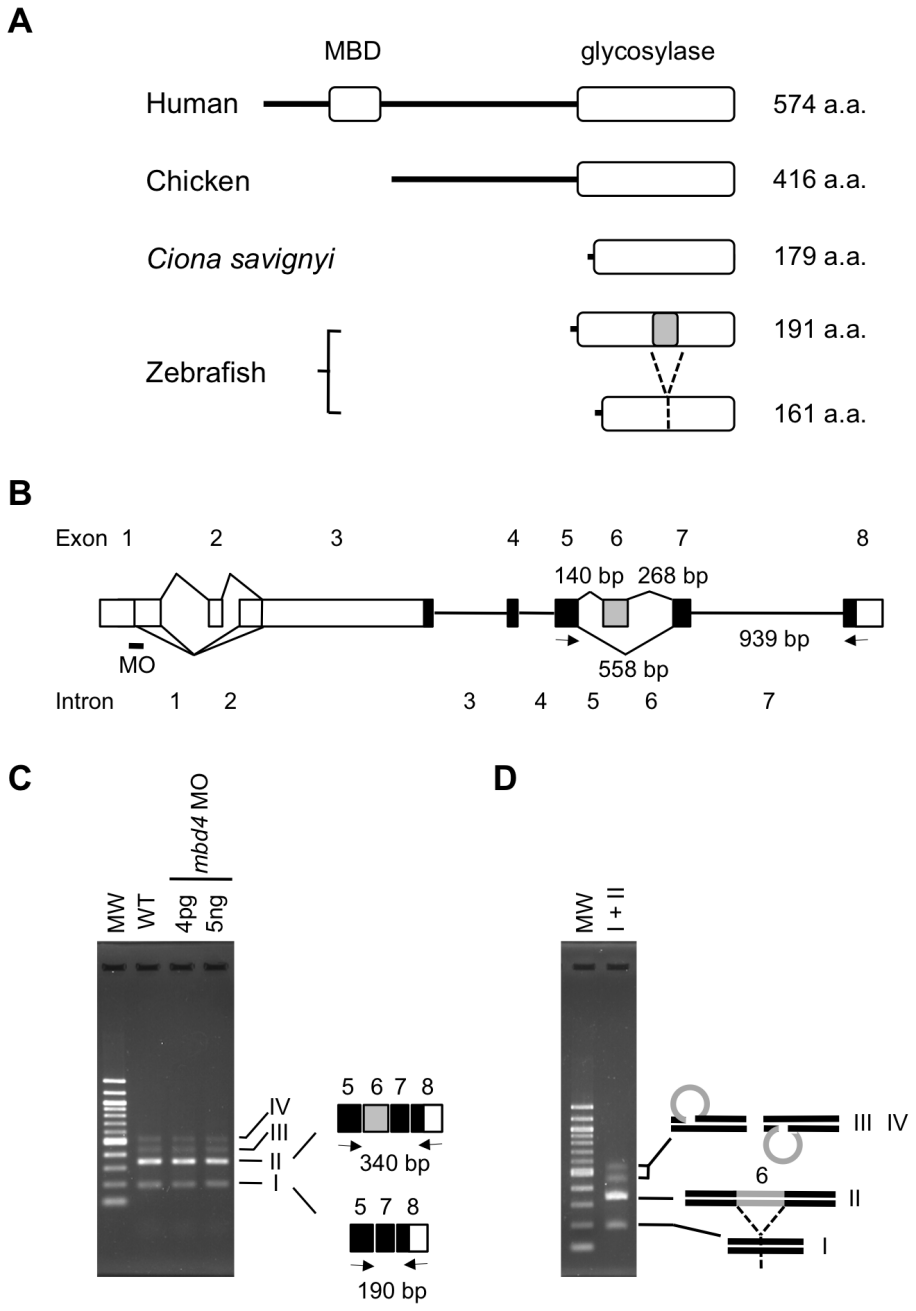
Zebrafish MBD4 lacks a MBD and *mbd4* MO did not interfere splicing of transcripts. (A) Schematic structures of MBD4 proteins in the chordata are indicated. The predominant form of MBD4 had MBD and a glycosylase domain near the N- and C- termini, respectively, similar to human MBD4. As rare cases in chordate, such as Chicken and Ciona, two zebrafish MBD4 proteins generated via alternative splicing lacked MBD. The shorter isoform lacked thirty amino acids, which corresponded to exon 6 (marked in grey). (B) The schematic structure of the zebrafish *mbd4* gene. Boxes show exons, and black and white boxes show translated and untranslated regions, respectively. Alternative splice events are indicated as diagonal lines. Five splicing variants of the 5′UTR and a shorter isoform that skipped exon 6 (grey box) were obtained by RT-PCR. The approximate positions of MO and primer pairs designed by Rai et al. [3] to detect aberrant splicing by MO are indicated by a small bar and arrows, respectively. The lengths of introns that may have been included in the RT-PCR product are shown. (C) With the primer set shown in (B), a region of *mbd4* cDNA was amplified from wild-type (WT) embryos in which the indicated amounts of *mbd4* MO were injected. The same banding pattern was observed irrespective of the MO injection. In addition to the expected band (II), three faint, but distinct bands (I, III, and IV) appeared both in wild-type and MO-injected embryos. The bottom band (I) was derived from an alternative splicing variant that skipped exon 6. (D) PCR amplification of band I and II in a single tube reproduced the same banding pattern on agarose gel as that in (C). The possible structures of bands III and IV are shown on the right of the gel.

In most vertebrates, MBD4 consists of two well-conserved and functional domains, an N-terminal methyl-CpG binding domain (MBD) and C-terminal DNA glycosylase domain, which are separated by a poorly conserved spacer region [9]. Rai et al. showed that the co-expression of zebrafish AID/Apobec and human MBD4 in zebrafish embryos led to the widespread DNA demethylation of the genome, including the retrotransposable elements, KenoDr1 and LINE-1, which are typically highly and constitutively methylated [3]. They suggested that the overexpression of human MBD4 with a MBD and a DNA glycosylase domain may account for the extended demethylation of the genome. Rai et al. used the human *mbd4* gene instead of the zebrafish *mbd4* in the co-expression experiment, and this may have been because the 5′ end of zebrafish *mbd4* cDNA had not been determined at that time [3]. Therefore, in their previous study, it was assumed that zebrafish MBD4 had a MBD, similar to human MBD4, and zebrafish MBD4 was regarded as being interchangeable with human MBD4 for demethylation. MBD4 in other fish species such as Coelacanth, Fugu, medaka, cave fish, Tilapia, Stickleback, and cod all have a MBD at the N-termini (http://www.ensembl.org/info/about/species.html). However, using 5′RACE, we found that the complete cDNA structure of the sole zebrafish homologue of human *mbd4* only coded for a DNA glycosylase domain and lacked a MBD domain (DDBJ accession number: AB918737), as were the rare cases for *Ciona* and chicken [23], [24] (http://www.ensembl.org/info/about/species.html) (Fig. 4A, see also S3 Fig.). An in-frame stop codon and eleven repetitive sequences were detected upstream of the first ATG of the open reading frame; therefore, there may be no additional coding sequences upstream of the first ATG (S3 Fig.). The most recent zebrafish genome-build (Zv9) remains incomplete at the *mbd4* locus, whereas some contigs of Illumina-generated sequences in the Sanger database (http://www.sanger.ac.uk/resources/zebrafish/genomeproject.html) that included regions upstream of *mbd4* revealed the absence of a sequence for the hidden MBD of this gene. Furthermore, although we attempted to identify a DNA sequence that may code for MBD using the MBD of mouse MBD4 as a query, only MBD1, MBD3, and MeCP2 (methyl-cytosine binding protein 2) were retrieved.

In addition to the lack of a MBD-coding sequence, zebrafish *mbd4* was unique in that alternative splicing produced a minor isoform that lacked exon 6 (150 bp, 50 amino acids), a middle region of the conserved DNA glycosylase domain (Fig. 4, see also S3 and S4A Figs.). It is currently not known whether the two forms of zebrafish MBD4, especially the smaller one, exhibit DNA glycosylase activity or function in combination or independently. Given the lack of MBD in zebrafish MBD4, together with the presence of two forms of the protein in zebrafish embryos, it would be premature to suggest that the functions of zebrafish MBD4 proteins could be deduced from the results obtained with the overexpression of full-length human MBD4 in zebrafish embryos.

### *mbd4* MO was non-functional, whereas *aid* MO blocked its own transcription

**Figure 5:**
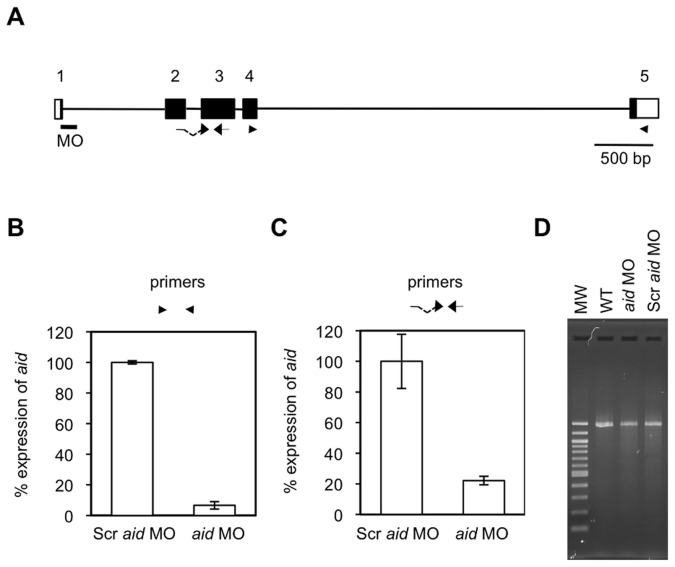
*aid* MO suppressed the expression of the *aid* gene. (A) The genomic structure of the *aid* gene was drawn, as in Fig. 4 (B). (B) qPCR analyses of the *aid* transcripts from Scr *aid* MO and *aid* MO-injected AB/Tü embryos were conducted by the primer set shown as arrowheads in (A). Scr *aid* MO is a negative control for *aid* MO that has a scrambled sequence for *aid* MO. (C) A MO-induced reduction in the expression of *aid* was confirmed with Tü line by qPCR with a primer set that differed from the primer set used in (B), and was shown as arrows in (A). Gene expression was normalized against *rpl13a*, and data represent the mean ± standard error of the mean (s.e.m.) from three independent experiments. (D) cDNAs derived from wild-type (WT) and MO-injected embryos were used for 5′RACE of *EF1a1*. The products were checked as in (B).

To knockdown the activities of MBD4 and AID, Rai et al. designed MOs against the boundaries between the first exon and first intron of the *mbd4* or *aid* gene to inhibit the correct splicing of their transcripts (Figs. 4B and 5A), and confirmed their efficacy by detecting their unspliced transcripts [3]. To confirm these findings, we injected two different amounts of AID and MBD4 MOs, 4pg and 5ng, the former of which was used in their previous study [3]. Since 4pg of MO was approximately three orders of magnitude lower than the amount of MO commonly used [5], [25], [26], we also used 1,000 times more of the former, 5ng of MO. We then attempted to detect missplicing of the *mbd4* or *aid* transcript in *mbd4* or *aid* MO-injected embryos, respectively, with the same primer sets used in the previous study (Figs. 4B and 5A). When PCR amplified a region of *mbd4* cDNA that spanned exon 6 and 7, we encountered two unanticipated results. First, in addition to a band of the expected size of 340 bp (fragment II in Fig. 4C), three faint DNA fragments were generated from control embryos (Fig. 4C and S4A Fig.), in contrast to the one amplified fragment from control embryos in the previous study [3]. We cloned and sequenced all four fragments, and found that they may have been derived from two alternatively spliced mRNA: the bottom band of 190 bp (fragment I in Fig. 4C) was from cDNA that skipped exon 6 of the *mbd4* gene, the splicing variant, as described above. The second bottom band was from cDNA of 340 bp, which retained exon 6 (fragment II in Fig. 4C). The two other larger fragments, depicted as III and IV in Fig. 4C, appeared to be a heteroduplex of fragments I and II (Fig. 4D) because the sequences of cloned fragments III and IV were identical to either the sequence of fragment I or II. When we re-amplified fragments I and II in a single tube, four DNA bands again appeared on the agarose gel (Fig. 4D). Therefore, it was likely that the looped-out exon 6 region of heteroduplex DNA could be an obstacle for migration in agarose and the degree of delayed migration may further differ between the two forms of the heteroduplex, depending on the strand that is looped-out.

Second, the same levels of the four PCR products (fragments I, II, III, and IV) were amplified from *mbd4* MO-injected embryos (Fig. 4C), which indicated that the *mbd4* MO designed by Rai et al. [3] did not cause the missplicing of *mbd4* transcripts at the position indicated in the previous study at either 4 pg or 5ng. We doubted whether the position of PCR amplification that spanned exon 6 and 7 would be adequate to detect missplicing because it was located apart from the MO position (exon 1/intron 1 boundary). Targeting a splice junction of the first exon in pre-mRNA by MO generally triggers the complete or partial inclusion of intron 1 [26], [27], [28]. Therefore, we PCR amplified a region that spanned introns 1 and 2 of the *mbd4* gene, and still could not detect any aberrant splicing variants (S4B Fig.). These results indicated that *mbd4* MO did not exert splicing blocker activity at the region specified in the previous study or at the exon 1/intron 1 boundary. Thus, whether zebrafish MBD4 is involved in demethylation has yet to be confirmed.

*aid* MO was also designed to anneal the first exon and first intron boundary (Fig. 5A) and reportedly caused missplicing [3]. However, we demonstrated by qPCR analyses that the injection of *aid* MO suppressed the expression of *aid* even at a very low amount (4 pg) in AB/Tü strain (Fig. 5B). The primer set used was designed for the amplification of exons 4 and 5 of *aid* (Fig. 5A); therefore, it is unlikely that intron 1 (870 bp), which may be left unspliced by *aid* MO, hampered PCR. In accordance with this result, qPCR analyses with a different set of primers that spanned intron 2 revealed a marked decrease in the amount of *aid* transcription in *aid* MO-injected Tü embryos (Fig. 5C). The 5′RACE products of elongation factor 1a1 (*EF1a1*) could be obtained not only from wild-type, but also from *aid* MO-injected AB/Tü embryos, which showed that our inability to amplify *aid* cDNA was not due to the degradation of mRNA extracted from *aid* MO-injected embryos (Fig. 5D). Since the binding site of *aid* MO was close to the 5′ end of the *aid* transcript (50∼60 bp downstream of the transcription start site), *aid* MO may prevent the progression of transcription. Although *aid* MO did not induce aberrant splicing as reported, *aid* MO may have suppressed the function of the AID protein in zebrafish embryos.

### No gain in methylation at the CpG islands of *neurod2*, *sox1a*, or *atoh1a* by *aid* or *mbd4* MO

**Figure 6:**
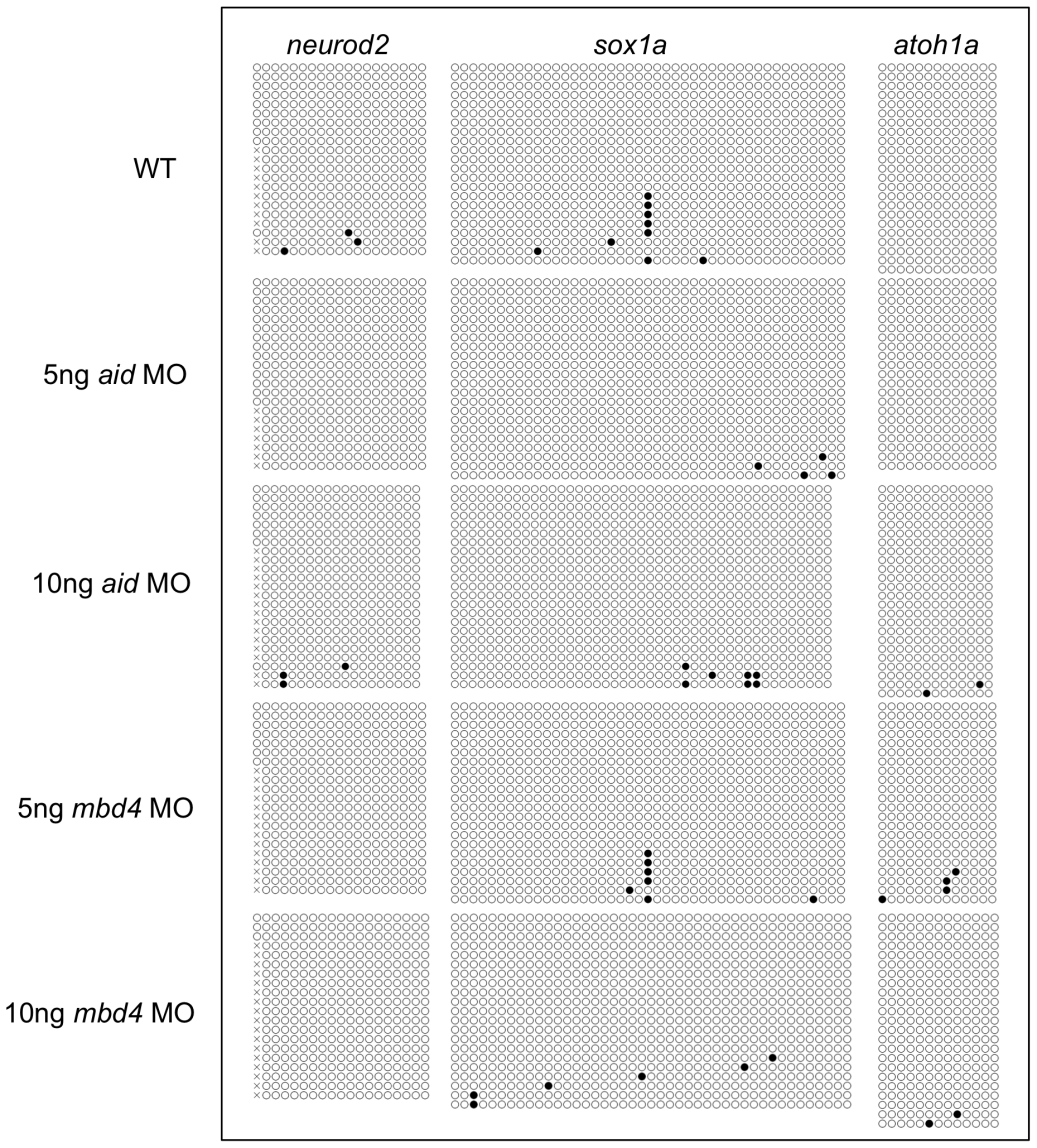
Neither *aid* MO nor *mbd4* MO elicited methylation at the CpG islands of neuronal genes. Bisulfite sequence analysis was used to examine the methylation of the CpG islands in the three genes indicated. Black and white circles are methylated and unmethylated cytosines, respectively. Crosses denote the positions at which CpG was absent due to polymorphisms.

Based on the finding that the knockdown of AID, Gadd45a, or MBD4 by MO caused the loss of neuronal gene expression at 24 hpf, Rai et al. compared the methylation status of transcription factor genes involved in neurogenesis at the CpG islands in *aid* or *mbd4* MO-injected embryos with those in control MO-injected embryos at 80% epiboly [3]. Using methylated DNA immunoprecipitation (MeDIP) alone or in combination with bisulfite sequence analyses, Rai et al. reported that a pronounced increase in CpG methylation at the CpG islands of *neurod2*, *sox1a,* and *atoh1a* genes following the injection of *aid* MO or *mbd4* MO [3]. To confirm the reproducibility of these findings, we repeated the experiment three times by two different operators; one operator used 5 ng and 10 ng of *aid* and *mbd4* MO (Fig. 6) and the other used four different amounts of *aid* and *mbd4* MO (4 pg, 10 pg, 5 ng, 10 ng) for AB/Tü (S5 Fig.) and 5 ng of *aid* and *mbd4* MO for Tü (S6 Fig.). However, bisulfite sequence analysis in each case revealed no increases in methylation at the CpG islands at any amounts of the two MOs. The absence of an increase in methylation by *mbd4* MO was consistent with *mbd4* MO being non-functional. In contrast, we consider it unlikely that AID is involved in maintaining the hypomethylated status of these CpG islands because *aid* MO severely attenuated *aid* transcription, as described above.

## Discussion

Rai et al. previously reported demethylation activity in zebrafish embryos that was catalyzed by the concerted actions of AID, MBD4, and Gadd45 [3]. They later demonstrated that demethylation activity regulated intestinal cell fating [17]. To the best of our knowledge, no other studies have confirmed these findings since their publication; therefore, we repeated the experiments performed in the former study. Rai et al. showed that the injection of M-DNA into zebrafish embryos caused the demethylation of both genomic DNA and the injected DNA fragment itself [3], a unique response whose biological significance remains unclear. This finding was the rationale for the presence of demethylation activity in zebrafish embryos, and the phenomenon was utilized as an assay system to uncover the involvement of three proteins, AID, MBD4, and Gadd45, in demethylation [3]. Therefore, we first examined the M-DNA injection into zebrafish eggs, but were unable to confirm the phenomenon even though the same M-DNA fragment was used. To detect the M-DNA-dependent global demethylation of the zebrafish genome, we adopted one of the two procedures used in their previous study: the digestion of genomic DNA with the methylation-sensitive enzyme HpaII, together with HpyCH4IV, an enzyme that has been used to sensitively detect age-related hypomethylation in the zebrafish genome [1]. Qualitative analysis with restriction enzymes was sufficiently sensitive to detect the small decrease that occurred in global methylation in *dnmt1* MO-injected embryos. In addition, we employed bisulfite sequencing to detect DNA methylation changes at specific loci. We, however, did not detect demethylation of M-DNA or several genomic loci, both of which were examined in our study.

Conflicting results were further obtained regarding gene induction by M-DNA. Rai et al. showed that the expression of deaminase family genes, including *aid*, *apobec2a*, and *apobec2b*, was clearly induced by the introduction of M-DNA into zebrafish embryos, and that these genes were involved in demethylation [3]. However, we did not observe any significant increases in the expression of *aid* or *apobec2a* genes, except for *apobec2b*. We cannot explain these discrepancies between our results and the findings reported by Rai et al [3]. Our inability to detect any signs of the demethylation or induction of *aid* or *apobec2a* raises serious concerns about the reality of M-DNA-related phenomena described in their previous study [3].

The presence of active demethylation in early development of the zebrafish remains controversial. Recent analyses of the whole-genome bisulfite sequence in zebrafish embryos until the germ ring stage (5.7 hpf) revealed that no loci were subjected to active demethylation [18], [19]. While these studies identified distinct loci in the maternal genome in which demethylation proceeds between 1-cell and 16/32-cell stages, demethylation is considered to be passive based on the gradual and apparently replication-dependent dilution process [18], [19]. Alternatively, as suggested by Rai et al., CpG islands, which were generally hypomethylated regardless of gene expression [29], may be under surveillance of active demethylation, which removes aberrant methylation from CpG islands [3]. However, some neurogenesis-related CpG islands shown to be methylated by *aid* MO in the previous study remained hypomethylated regardless of the suppression of *aid* transcription. We once again cannot account for this discrepancy, and it remains to be determined whether CpG islands in zebrafish are guarded by demethylation activity against *de novo* methylation.

Rai et al. showed that *mbd4* MO also caused the *de novo* methylation of some neurogenesis-related CpG islands, similar to *aid* MO [3]. However, we could not verify these findings, even when the amount of MOs used (10 ng) was 2500 times higher than those in the previous study (4 pg). Therefore, *mbd4* MO, which was used in the previous study, may not have been appropriate for testing the involvement of zebrafish MBD4 in demethylation because aberrant splicing was not induced by *mbd4* MO. Further analyses are clearly required to determine whether zebrafish MBD4 is related to demethylation.

Rai et al. suggested that the demethylation complex consisting of AID, MBD4, and Gadd45 could be recruited to 5mC by MBD in MBD4 [3]. In contrast to the assumption by Rai et al. [3], we found that the zebrafish MBD4 protein lacked a MBD. This result, together with the finding that AID or Gadd45 had no DNA binding motif, makes it difficult to explain how the proposed demethylation complex consisting of AID, MBD4, and Gadd45 could be recruited to 5mC. Thus, we did not examine the overexpression of zebrafish AID or zebrafish MBD4 in zebrafish eggs. Although Rai et al. reported that the forced co-expression of zebrafish AID and human MBD4 elicited demethylation [3], the possibility that the heterogeneous combination of the two proteins exerted artificial demethylation activity in zebrafish embryos cannot be ruled out because human MBD4 possesses a MBD.

A recent study revealed that zebrafish AID was unique among all orthologs because it was shown to efficiently deaminate 5mC *in vitro* [6]. However, the knockdown of *aid* in our experiments did not induce global methylation changes in the zebrafish genome at 5 or 13 hpf, which suggested that AID-dependent active demethylation in zebrafish may be loci-specific if it occurs at embryonic stages. The requirement of AID for DNA demethylation has not yet reached consensus, even in mouse systems; it has been supported by the findings of studies performed using primordial germ cells [30] and pluripotent ES/iPS cells [31], [32], [33], but not in those using activated B cells [34] in which there is adequate amounts of AID to mutate a number of non-*Ig* loci [35].

Given that AID is involved in active demethylation, another unresolved issue was the substrate of AID for demethylation. Recent findings on the TET DNA dioxygenase, which oxidized 5mC to 5-hydroxymethylcytosine (5hmC), suggest that 5hmC, but not 5mC could be a substrate of AID for demethylation [36]; 5hmC is deaminated by AID/APOBECs to produce 5-hydroxymethyluracil (5hmU), which can then be excised by 5hmU glycosylases and finally replaced with an unmethylated cytosine by base excision repair. The zebrafish genome encodes all three members of the TET family proteins, each of which converts 5mC to 5hmC [25]. However, 5hmU was not detected in mouse neuronal or HEK293 cells [36], [37], and recent *in vitro* findings showed that the deamination of 5hmC by AID was unlikely to occur [38], [39]. These studies challenge the plausibility of the proposed pathway that invoked 5hmC deamination in DNA demethylation.

We consider two points to be critical in order to confirm the reproducibility of the findings reported by Rai et al [3]: the demethylation of both zebrafish genomic DNA and M-DNA itself by the injection of M-DNA into zebrafish eggs, and the aberrant methylation at certain CpG islands due to *aid* MO or *mbd4* MO. We were unable to duplicate either of these points, and the reasons for the discrepancies between our results and the findings reported by Rai et al. [3] are unknown. It is important to note that the procedures used for the experiments described above were straightforward and well established, and the materials used were all purchased from manufacturers, except the zebrafish embryos, which makes it unlikely that we overlooked changes that should have been induced by M-DNA or MOs.

Taken together, we obtained the following results that were contradictory to previous studies: 1) demethylation was not detected following the injection of a methylated DNA fragment, 2) zebrafish MBD4 lacked a MBD, 3) *mbd4* MO was ineffective, and 4) *aid* MO was effective, but did not elicit methylation. These results, together with the absence of any actively-demethylated loci in the genome of early zebrafish embryos [18], [19], indicate that there is no evidence yet not only for AID/MBD4-coupled active demethylation in zebrafish embryos, but also for the presence of demethylation activity during the development of zebrafish.

## Conclusions

Our results raise serious concerns regarding the previously proposed model involving the concerted action of AID, MBD4 and GADD45 in active DNA demethylation in zebrafish.

## Materials and Methods

### Ethics statement

All animal experiments were conducted in strict accordance with relevant national and international guidelines: ‘Act on Welfare and Management of Animals’ (Ministry of Environment of Japan) and all steps were taken to minimize animal discomfort. Zebrafish embryos were euthanized by overdose with Tricaine. Ethics was approved from the Hiroshima University Animal Research Committee (Permit Number: F13-1).

### Zebrafish care

Wild-type (AB/Tü and Tübingen lines) zebrafish were maintained at 28.5°C in a 14 hr light: 10 hr dark cycle in our fish facility.

### Preparation of methylated DNA (M-DNA) and genomic DNA

To prepare M-DNA, a 736 bp-DNA fragment was PCR amplified from the luciferase gene in pGL3-SV40 (Promega), as described previously [3]. The PCR fragment was purified with QIA quick mini-spin columns (Qiagen) and methylated *in vitro* with HpaII methylase (New England Biolabs) according to the manufacturer's instructions. The resultant M-DNA was purified with phenol/chloroform/isoamyl alcohol followed by precipitation with ethanol. Prior to the injection, M-DNA was diluted in Danieau solution [58 mM NaCl, 0.7 mM KCl, 0.4 mM MgSO_4_, 0.6 mM Ca(NO_3_)_2_, 5.0 mM HEPES, pH7.6] [40] and it was injected at 200 pg per embryo. To prepare genomic DNA, zebrafish embryos were harvested at the designated time points and DNA was extracted using Proteinase K and SDS [40]. M-DNA and genomic DNA were dissolved in 10 mM Tris-HCl (pH 8.0)/0.1 mM EDTA, and M-DNA and genomic DNA concentrations were measured on a NanoDrop 2000 spectrophotometer (NanoDrop Technologies Inc., ThermoFisher Scientific).

### Digestion of M-DNA and genomic DNA by restriction enzymes

Fifty nanograms of M-DNA was digested with either MspI (5 units) or HpaII (5 units) for 2 hr at 37°C. One hundred nanograms of genomic DNA was digested with MspI (10 units), HpaII (10 units), or HpyCH4IV (10 units) for 8 hr at 37°C. Uncut, HpaII cut, and MspI cut M-DNA were then separated on a 2% agarose gel with a 100-bp DNA ladder marker. Uncut, MspI cut, HpaII cut, and HpyCH4IV cut genomic DNA were separated on a 1.2% agarose gel with a 1-kb DNA ladder marker. Gels were stained with ethidium bromide (0.5 mg/ml) and then destained in water. DNA-size markers and restriction enzymes were purchased from New England Biolabs, except for the 100-bp ladder maker in Fig. 5B (Invitrogen).

### Morpholino (MO) Injections

The MO sequences used were identical to those used by Rai et al. [3]. MOs obtained from Gene-tools LLC Ltd. (Philomath, OR) were dissolved in water, and the concentrations of dissolved MOs were determined by the procedure shown on the home page of Gene-tools (http://www.gene-tools.com/) using a NanoDrop 2000 spectrophotometer. Prior to the injection, MOs were diluted in Danieau solution [40]. M-DNA or MO was injected into zebrafish eggs at the one-cell stage and ten embryos were then used to extract genomic DNA.

### Bisulfite sequence analyses

In the methylation analysis for zebrafish genes and M-DNA, 200 ng of genomic DNA was processed using the EZ DNA Methylation-Gold Kit (Zymo Research, Orange, CA, USA) to convert unmethylated cytosines into uracils. Injected M-DNA was recovered from ten embryos with genomic DNA at different time points. The primer pairs used to amplify *neurod2*, *sox1a*, and *athoh1a* CpG islands were the same as those used in the previous study [3]. We used the MethPrimer program to choose the sequences of the other primer pairs for bisulfite sequencing [41]. Primer sequences and PCR conditions are shown in S1 Table. PCR fragments were cloned into the pGEM-T Easy vector (Promega, Madison, WI, USA) and were used to transform the *Escherichia coli* DH5alpha strain. Plasmids isolated from 24 and 12 transformants for zebrafish genes and M-DNA, respectively, were sequenced with the DYEnamic ET Terminator Cycle Sequencing kit (GE Healthcare, Piscataway, NJ, USA) on an ABI3100 genetic analyzer (Applied Biosystems, Foster City, CA, USA). QUMA was used to analyze sequence data and drawing figures [42].

### Preparation of total RNA and first strand cDNA synthesis

To extract total RNA from embryos, pools of 20 live embryos were homogenized in 500 µl TRIzol Reagent (Invitrogen, Carlsbad, CA, USA) using a Kontes Pellet Pestle in companion tubes (Thermo Fisher Scientific, Waltham, MA, USA). Extracted RNA was then purified by an RNeasy Mini kit (QIAGEN, Hilden, Germany). The quantity of total RNA was measured on a NanoDrop 2000 spectrophotometer. Five hundred nanograms of total RNA was mixed with an RNA loading buffer containing 0.4 mg/ml ethidium bromide, and electrophoresed on a 1.0% formaldehyde-containing agarose gel to check the quality of RNA. One µg of total RNA from embryos at 4 hpf, 13 hpf, and 28 hpf fish was reverse-transcribed in a total volume of 20 µl to produce cDNA using the High Capacity cDNA Reverse Transcription kit (Applied Biosystems, Foster City, CA, USA). In all cases, a reverse-transcriptase negative control was used to test genomic DNA contamination.

### Quantitative real-time RT-PCR (qPCR)

Standard reactions for qPCRs were prepared as follows: 10 µl of SsoFast EvaGreen Supermix (BioRad, Hercules, CA, USA), 0.25 µl of the forward and reverse primers (20 µM each), 0.67 µl of the template, and 8.83 µl of water. Templates were 1∶20 diluted cDNA samples, and in the case of negative controls, cDNAs were replaced by water. All real time assays were performed in triplicate using a LightCycler (Roche Diagnostics, Mannheim, Germany). After denaturing samples at 95°C for 30 s, forty amplification cycles were performed, with each cycle consisting of 95°C for 5 s followed by 59°C for 20 s. Primer information is shown on S1 Table. *P-values* were calculated using an unpaired *t* test.

### RT-PCR and 5′RACE

Using first strand cDNA as a template, *aid* and *mbd4* were PCR amplified either with a set of primers designed by Rai et al. [3] or with primers designed in this study. 5′RACE was performed using a SMART RACE cDNA Amplification Kit (Clontech) following the manufacturer's instructions. PCR products were run on a 2% agarose gel, except for the RT-PCR product of *mbd4* (exon1∼exon3), which was separated on a 10∼20% gradient polyacrylamide gel (Wako, Japan).

## Supporting Information

S1 Fig **Absence of genome demethylation following the injection of a methylated DNA fragment into zebrafish eggs of Tü line.** (A) Undigested genomic DNA of control (W), M-DNA-injected (M), and *dnmt1* MO-injected (MO) embryos at the indicated time points (hours post fertilization; hpf) were run on an agarose gel. (B, C, D) The same genomic DNAs as those used and shown in (A) were digested with MspI (B), HpaII (C), or HpyCH4IV (D), and run on an agarose gel. The molecular weight markers of DNA loaded on the first lanes of gels, shown as MW, were 1 kb ladders (A, B, C, and D). Note that smearing down of high-molecular DNA, thereby reducing the methylation level, was discernible only in the genomic DNA from *dnmt1* MO-injected embryos (white arrows in C and D). (E) Bisulfite sequencing revealed no significant changes in the methylation levels of injected M-DNA recovered from 10 Tü embryos at any of the indicated time points.
(TIF)

S2 Fig **M-DNA did not induce the expression of** ***aid*** **or** ***apobec2a*** **in Tü.** (A) The expression levels of AID/Apobec family genes and *mbd4* at the indicated time points were quantified by qPCR, and these expression levels in wild-type Tü embryos (white rectangles) were compared with those in M-DNA-injected Tü embryos (black rectangles). (B) The expression levels of Gadd45-family genes between wild-type and M-DNA-injected Tü embryos were compared using qPCR. Gene expression was normalized against *rpl13a* (*ribosomal protein L13a* gene) and data represent the mean ± standard error of the mean (s.e.m.) from three independent experiments; **P<0.01 and *P<0.05.
(TIF)

S3 Fig **Nucleotide and amino acid sequences of complete zebrafish** ***mbd4*** **cDNA.** Horizontal arrows indicate the eleven-fold repetition of the short homologous sequences in the 5′ UTR. An in-frame stop codon in the 5′ UTR is boxed. Vertical arrows show the positions of introns. Dotted arrows show the primers used to detect aberrant splicing in Fig. 4C. The dotted line corresponded to exon 6, which could be skipped by alternative splicing. *mbd4* MO designed by Rai et al. [3] was expected to hybridize with the underlined sequence in the 5′ UTR. The sequence presented here corresponded to a splicing variant form, a, in S2 Fig. A partial amino acid sequence homologous to this MBD4 sequence was deposited as “methyl-CpG-binding domain protein 4-like” in the NCBI database under the accession number XP_005169167.
(TIF)

S4 Fig **The absence of aberrant splicing at the exon 1/intron 1 boundary by** ***mbd4*** **MO.** (A) With the same primer set shown in Fig. 4, a region of *mbd4* cDNA was amplified from wild-type (WT) Tü embryos and the Tü embryos in which *mbd4* MO or scrambled (Scr) *mbd4* MO was injected. The same banding pattern was observed irrespective of the MO injection as in Fig. 4C. (B) The RT-PCR products of *mbd4* cDNA derived from wild-type (WT) and MO-injected embryos at 80% epiboly were run on a polyacrylamide gel, stained with ethidium bromide. The positions of the primers (arrows) and MO used were shown on the top right with 5′ UTR of *mbd4*. Note that exon1 and exon3 have two splicing donor and acceptor sites, respectively. The sizes and schematic structures of the five distinct bands are shown on the right. The three faint bands bracketed may be hetroduplexes of the splicing variants.
(TIF)

S5 Fig **Independent test for the effects of** ***aid*** **MO and** ***mbd4*** **MO on demethylation.** The methylation of CpG islands in the three genes indicated was examined by bisulfite sequence analyses, as in Fig. 6, except for the MO injection, which was performed by a different operator. Black and white circles are methylated and unmethylated cytosines, respectively. Crosses denote the positions at which CpG was absent due to polymorphisms.
(TIF)

S6 Fig **Neither** ***aid*** **MO nor** ***mbd4*** **MO elicited methylation at the CpG islands of neuronal genes in Tü line.** Bisulfite sequence analysis was used to examine the methylation of the CpG islands in the three genes indicated. Black and white circles are methylated and unmethylated cytosines, respectively. Crosses denote the positions at which CpG was absent due to polymorphisms.
(TIF)

S1 Table **Primers and PCR conditions for bisulfite genomic sequencing, quantitative RT-PCR, and RACE.**
(PDF)
