# Supplementary figures and images for: No Evidence for AID/MBD4-Coupled DNA Demethylation in Zebrafish Embryos
Source: PLoS One. 2014 Dec 23;9(12):e114816. doi: 10.1371/journal.pone.0114816 (PMC4275248; doi:10.1371/journal.pone.0114816)

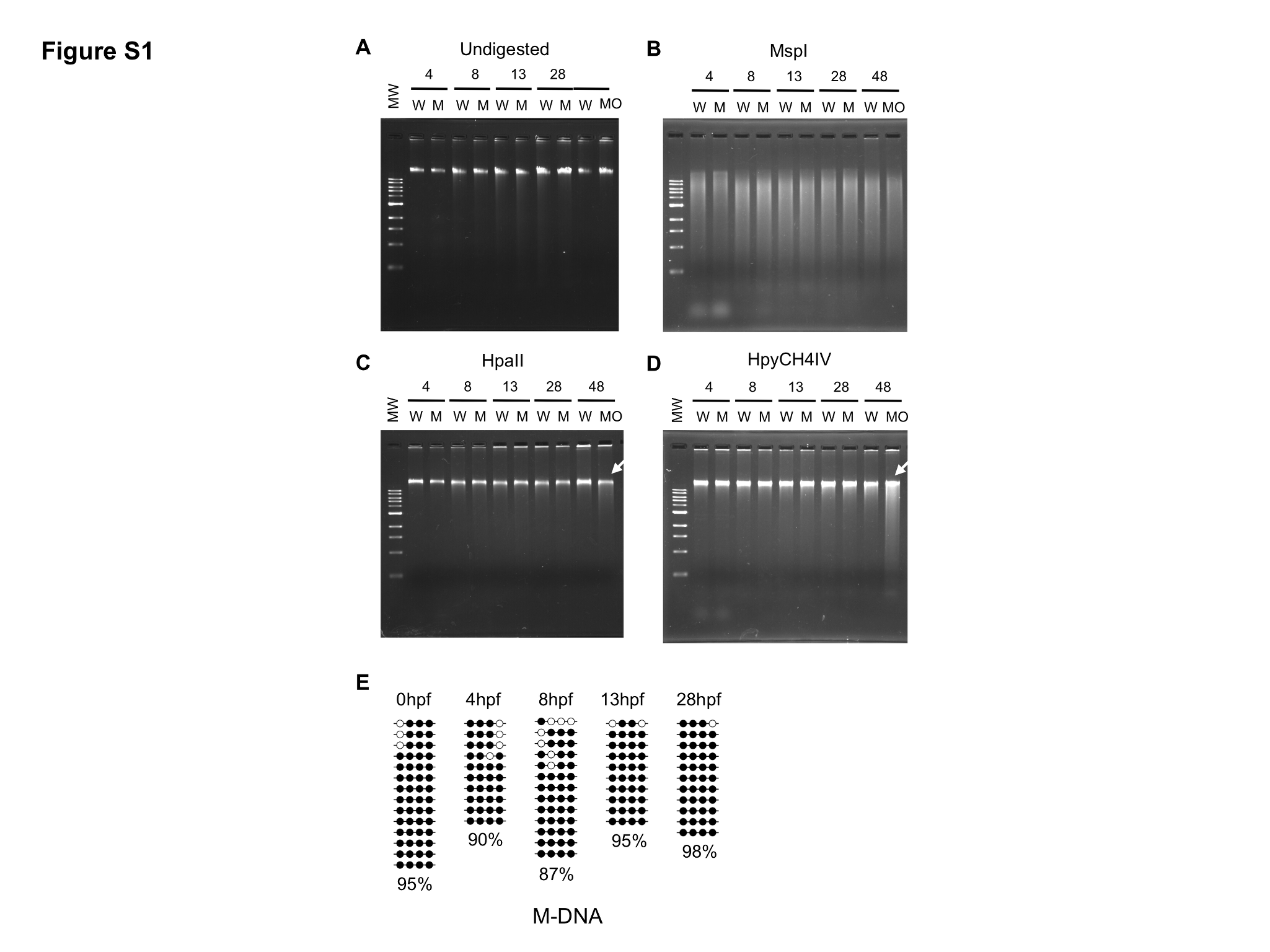

Supplement: S1 Fig — Absence of genome demethylation following the injection of a methylated DNA fragment into zebrafish eggs of Tü line. (A) Undigested genomic DNA of control (W), M-DNA-injected (M), and dnmt1 MO-injected (MO) embryos at the indicated time points (hours post fertilization; hpf) were run on an agarose gel. (B, C, D) The same genomic DNAs as those used and shown in (A) were digested with MspI (B), HpaII (C), or HpyCH4IV (D), and run on an agarose gel. The molecular weight markers of DNA loaded on the first lanes of gels, shown as MW, were 1 kb ladders (A, B, C, and D). Note that smearing down of high-molecular DNA, thereby reducing the methylation level, was discernible only in the genomic DNA from dnmt1 MO-injected embryos (white arrows in C and D). (E) Bisulfite sequencing revealed no significant changes in the methylation levels of injected M-DNA recovered from 10 Tü embryos at any of the indicated time points. (TIF) [file pone.0114816.s001.tif]

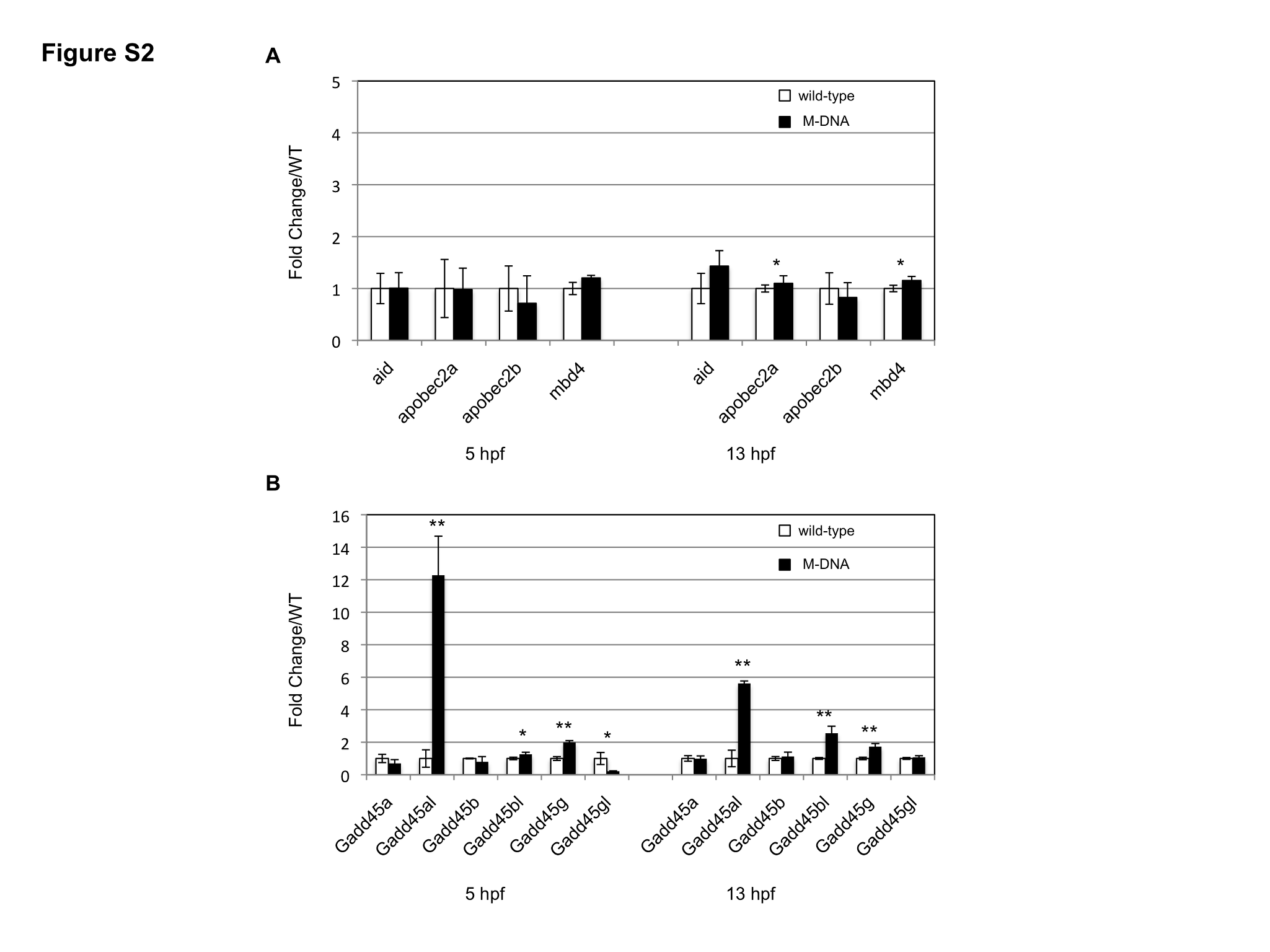

Supplement: S2 Fig — M-DNA did not induce the expression of aid or apobec2a in Tü. (A) The expression levels of AID/Apobec family genes and mbd4 at the indicated time points were quantified by qPCR, and these expression levels in wild-type Tü embryos (white rectangles) were compared with those in M-DNA-injected Tü embryos (black rectangles). (B) The expression levels of Gadd45-family genes between wild-type and M-DNA-injected Tü embryos were compared using qPCR. Gene expression was normalized against rpl13a (ribosomal protein L13a gene) and data represent the mean ± standard error of the mean (s.e.m.) from three independent experiments; **P<0.01 and *P<0.05. (TIF) [file pone.0114816.s002.tif]

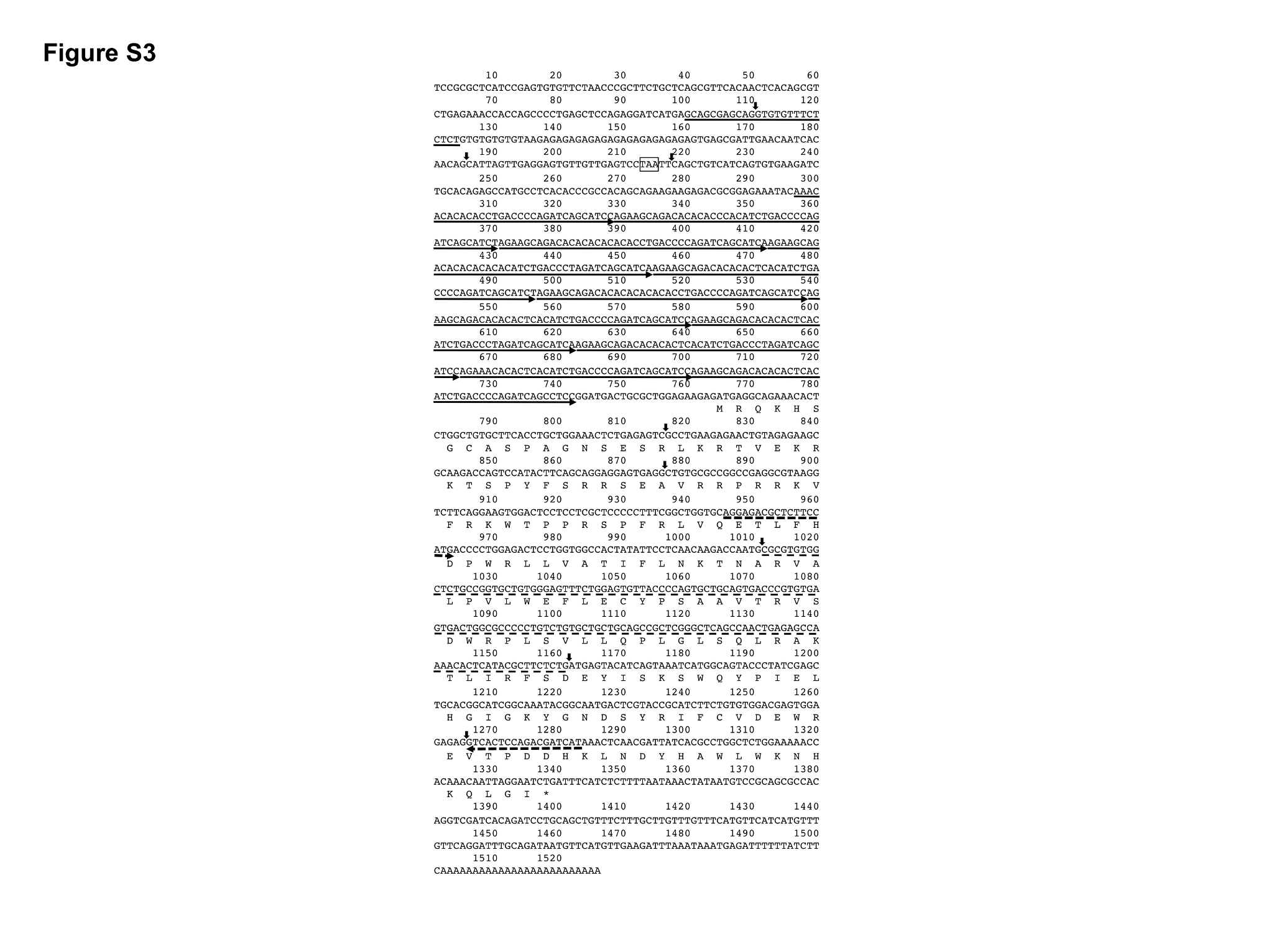

Supplement: S3 Fig — Nucleotide and amino acid sequences of complete zebrafish mbd4 cDNA. Horizontal arrows indicate the eleven-fold repetition of the short homologous sequences in the 5′ UTR. An in-frame stop codon in the 5′ UTR is boxed. Vertical arrows show the positions of introns. Dotted arrows show the primers used to detect aberrant splicing in Fig. 4C. The dotted line corresponded to exon 6, which could be skipped by alternative splicing. mbd4 MO designed by Rai et al. [3] was expected to hybridize with the underlined sequence in the 5′ UTR. The sequence presented here corresponded to a splicing variant form, a, in S2 Fig. A partial amino acid sequence homologous to this MBD4 sequence was deposited as “methyl-CpG-binding domain protein 4-like” in the NCBI database under the accession number XP_005169167. (TIF) [file pone.0114816.s003.tif]

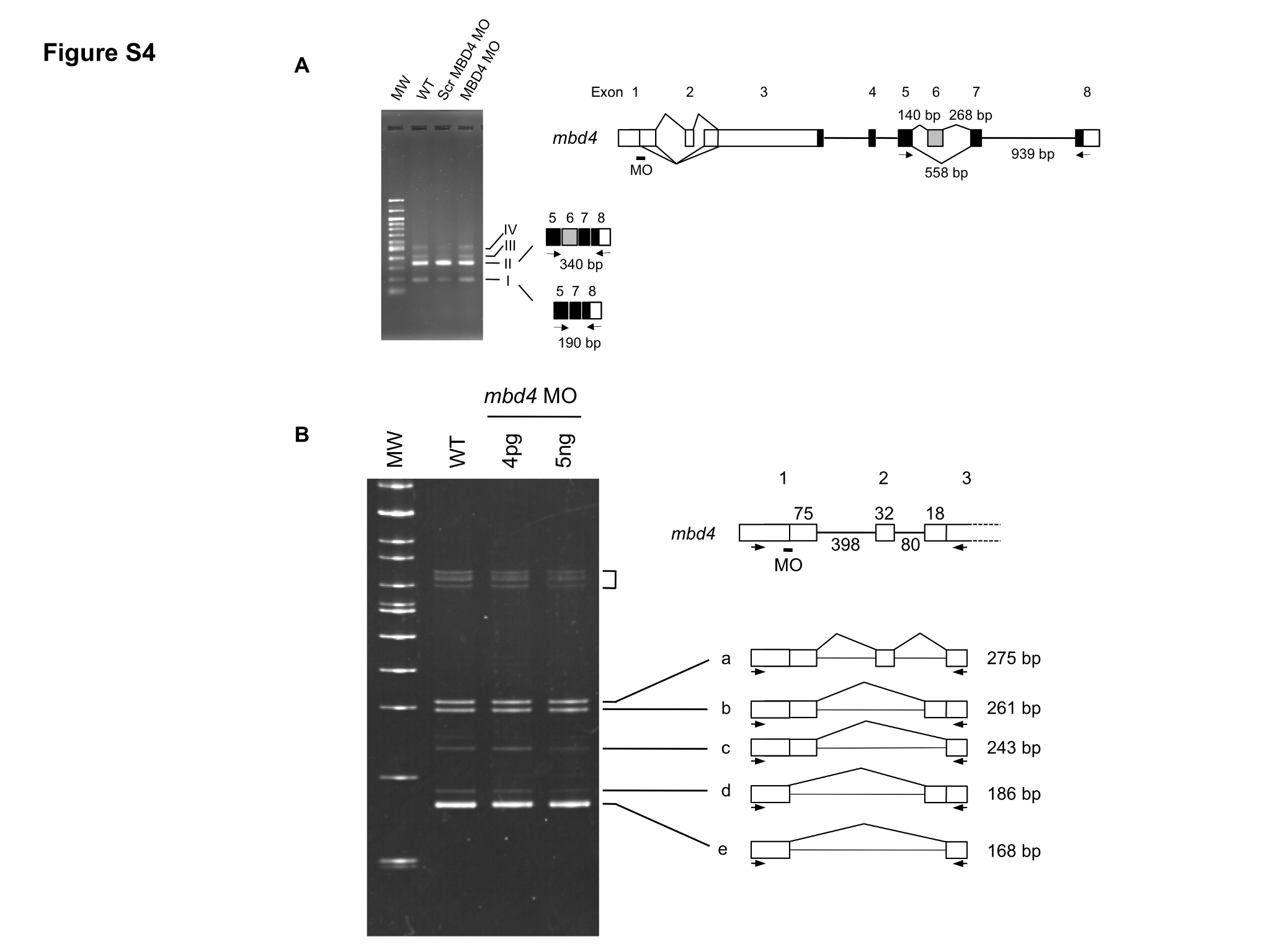

Supplement: S4 Fig — The absence of aberrant splicing at the exon 1/intron 1 boundary by mbd4 MO. (A) With the same primer set shown in Fig. 4, a region of mbd4 cDNA was amplified from wild-type (WT) Tü embryos and the Tü embryos in which mbd4 MO or scrambled (Scr) mbd4 MO was injected. The same banding pattern was observed irrespective of the MO injection as in Fig. 4C. (B) The RT-PCR products of mbd4 cDNA derived from wild-type (WT) and MO-injected embryos at 80% epiboly were run on a polyacrylamide gel, stained with ethidium bromide. The positions of the primers (arrows) and MO used were shown on the top right with 5′ UTR of mbd4. Note that exon1 and exon3 have two splicing donor and acceptor sites, respectively. The sizes and schematic structures of the five distinct bands are shown on the right. The three faint bands bracketed may be hetroduplexes of the splicing variants. (TIF) [file pone.0114816.s004.tif]

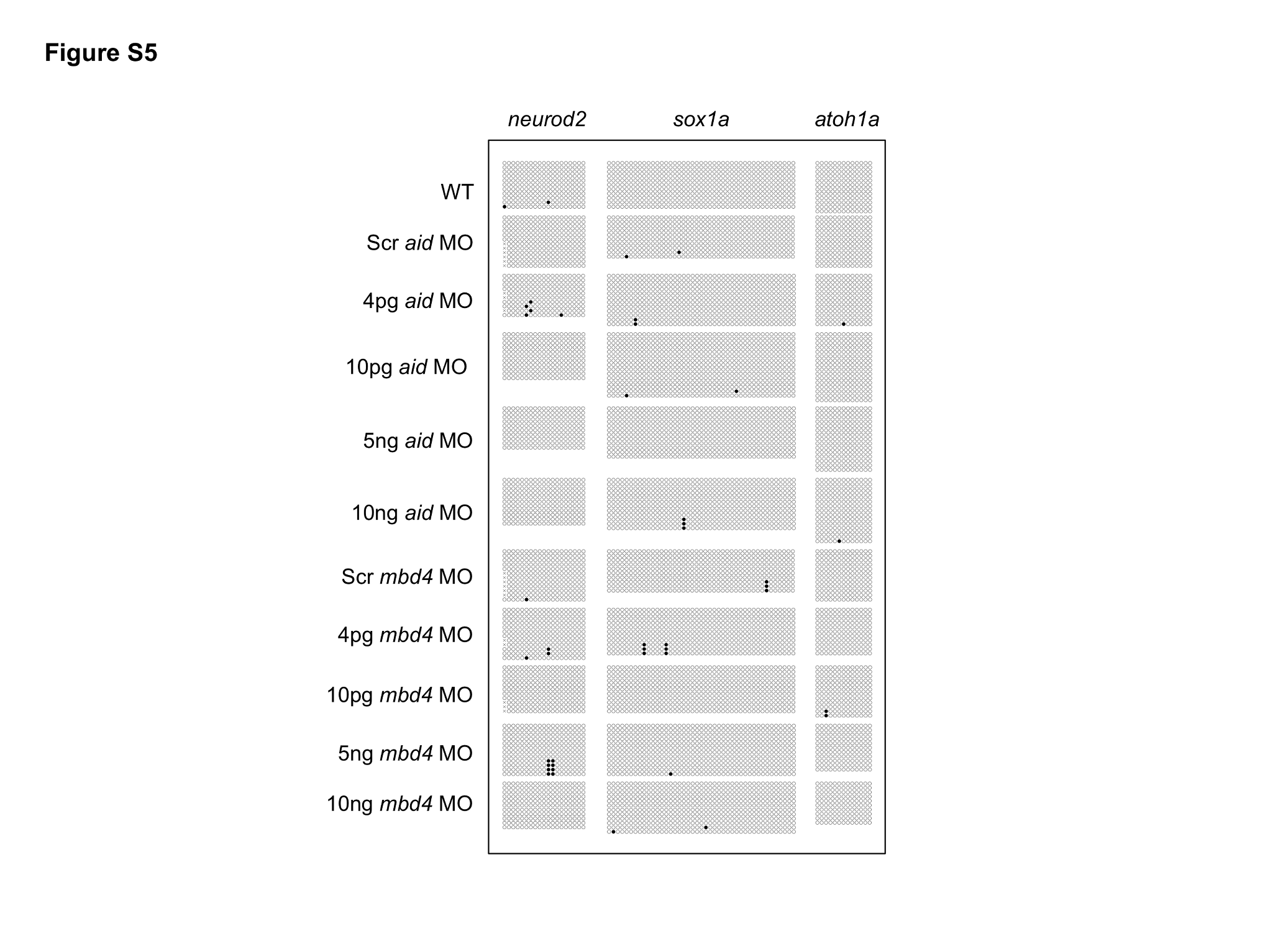

Supplement: S5 Fig — Independent test for the effects of aid MO and mbd4 MO on demethylation. The methylation of CpG islands in the three genes indicated was examined by bisulfite sequence analyses, as in Fig. 6, except for the MO injection, which was performed by a different operator. Black and white circles are methylated and unmethylated cytosines, respectively. Crosses denote the positions at which CpG was absent due to polymorphisms. (TIF) [file pone.0114816.s005.tif]

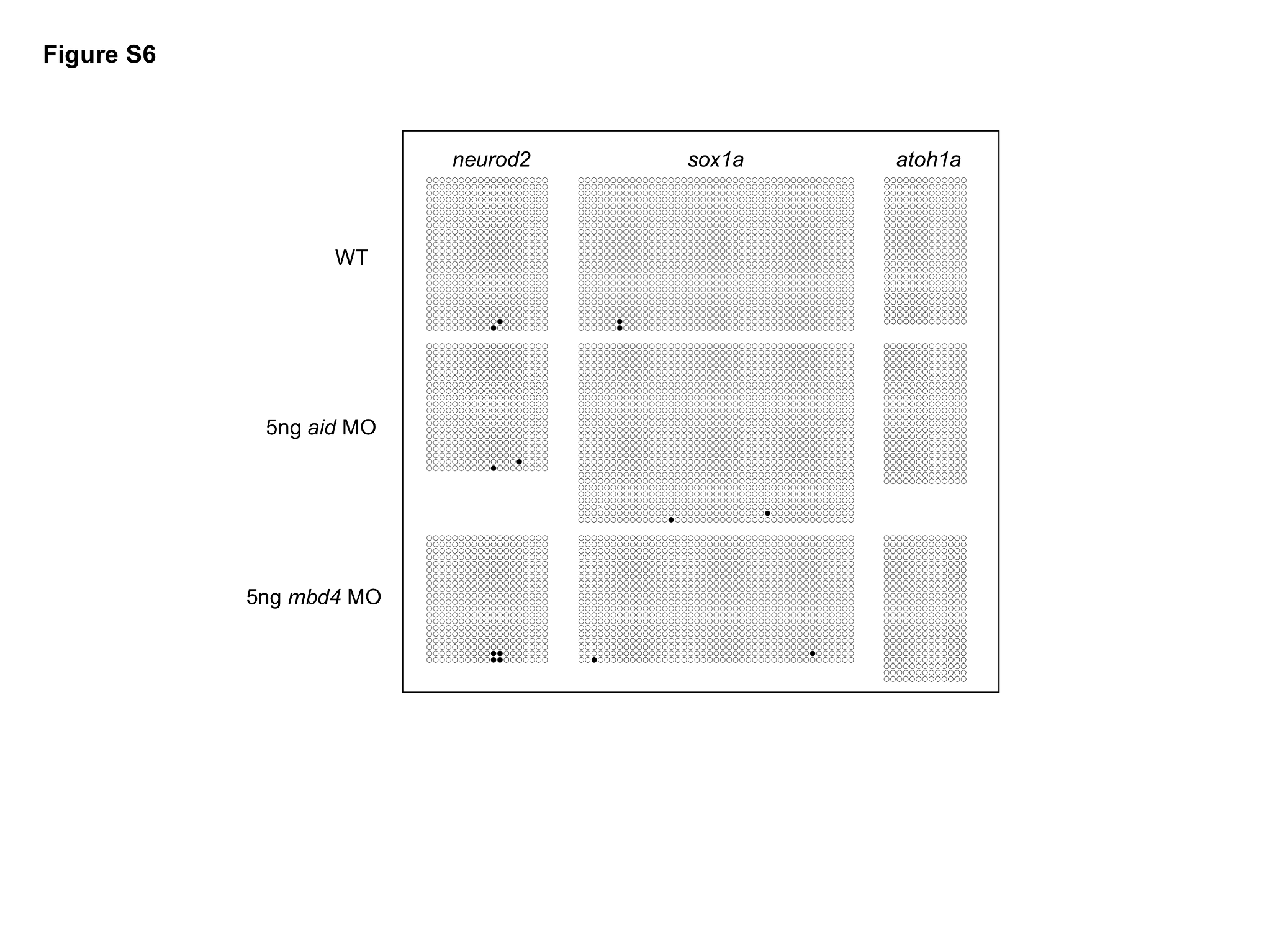

Supplement: S6 Fig — Neither aid MO nor mbd4 MO elicited methylation at the CpG islands of neuronal genes in Tü line. Bisulfite sequence analysis was used to examine the methylation of the CpG islands in the three genes indicated. Black and white circles are methylated and unmethylated cytosines, respectively. Crosses denote the positions at which CpG was absent due to polymorphisms. (TIF) [file pone.0114816.s006.tif]
