# Supplementary material for: No Evidence for AID/MBD4-Coupled DNA Demethylation in Zebrafish Embryos
Source: PLoS One. 2014 Dec 23;9(12):e114816. doi: 10.1371/journal.pone.0114816 (PMC4275248; doi:10.1371/journal.pone.0114816)
Supplement: S1 Table — Primers and PCR conditions for bisulfite genomic sequencing, quantitative RT-PCR, and RACE. (PDF) [file pone.0114816.s007.pdf]

Table S1 – Primers and PCR conditions for bisulfite genomic sequencing, quantitative RT-PCR, and RACE

Gene	Figures	Primer sequences	PCR conditions	CpGs	Product
M-DNA	1A	ATGGAAGACGCCAAAAACATAA	95ºC, 10 m:(94ºC, 30 s: 55ºC, 30 s:		736 bp
		CGTGATGGAATGGAACAACA	72ºC, 30 s)×38 cycles: 72ºC, 7 m		
M-DNA	2A	GATGTTAAAAATATAAAGAAAGGT	95ºC, 10 m:(94ºC, 30 s: 54ºC, 30 s:	4	718 bp
		AAACAACACTTAAAATCACAATAT	72ºC, 1 m)×40 cycles: 72ºC, 7 m		
SINE3-1a	2B	TTTAATTTGTGGTTTGTGTGAGTTT	95ºC, 10 m:(94ºC, 30 s: 55ºC, 30 s:	15	350 bp
		CCACCAATATACAACATCCACTTAA	72ºC, 30 s)×25 cycles: 72ºC, 7 m		
		AAGGGGATATTATATTGTTAGTGGG (nest)	95ºC, 10 m:(94ºC, 30 s: 55ºC, 30 s:	7	148 bp
		ATAAAAAAACTTTAACCAAAACACC (nest)	72ºC, 30 s)×25 cycles: 72ºC, 7 m		
DANA	2B	TAATATAAAAATAAAATAAAATAAATTTTG	95ºC, 10 m:(94ºC, 30 s: 50ºC, 30 s:	18	469 bp
		CACCAAAATATTTCAATACTTTCT	72ºC, 1 m)×35 cycles: 72ºC, 7 m		
fli1a	2B	GTTTTTAATGGGTTTTTGTATATTT	95ºC, 10 m:(94ºC, 30 s: 55ºC, 30 s:	11	292 bp
		CTCACAAAACTACTATTATCAACATAACTA	72ºC, 30 s)×35 cycles: 72ºC, 7 m		
runx1	2B	TTTTTGGGGG TTTAGGAGAG TAT	95ºC, 10 m:(94ºC, 30 s: 50ºC, 30 s:	14	210 bp
		CCCATATAAAACAAATTCTAATACCTTAAA	72ºC, 1 m)×40 cycles: 72ºC, 7 m		
5S rRNA	2B	TTTGGTAGTTGATTTAAATAGTTAA	95ºC, 10 m:(94ºC, 30 s: 53ºC, 30 s:	7	160 bp
		CTTACAACACCTAATATTCCCAAAC	72ºC, 30 s)×40 cycles: 72ºC, 7 m		
KenoDr1	2B	GGTTTGTATTGAATTATTGGT	95ºC, 10 m:(94ºC, 30 s: 55ºC, 30 s:	16	282 bp
		AAATCATTTTCCTTAAAAATCA	72ºC, 30 s)×40 cycles: 72ºC, 7 m		
LINE-1	2B	GGTTGGAGATGGTTTATTTT	95ºC, 10 m:(94ºC, 30 s: 55ºC, 30 s:	50	551 bp
		TTCCTAAACCACAAATATAAACAT	72ºC, 30 s)×33 cycles: 72ºC, 7 m		
aid	3A, 5C	TCAGATTCTGCAGCCTTGC	95ºC, 30s:(94ºC, 5 s: 59ºC, 20 s)		136 bp
		CAGCACCATTTCTCTTCATCC	×40 cycles		
apobec2a	3A	CACAACGCACCCAAGTGA	95ºC, 30s:(94ºC, 5 s: 59ºC, 20 s)		227 bp
		CCTGTGATGGTCTCGAATG	×40 cycles		
apobec2b	3A	ATGGCAGACAAAAAGGACAG	95ºC, 30s:(94ºC, 5 s: 59ºC, 20 s)		287 bp
		CGGTCTCCTACAATGATCTC	×40 cycles		
mbd4	3A	AAGAGACGCTCTTCCATGA	95ºC, 30s:(94ºC, 5 s: 59ºC, 20 s)		340 bp
		TTATGATCGTCTGGAGTGAC	×40 cycles		
gadd45a	3B	CGCTTAATGTGGACCCAGAC	95ºC, 30s:(94ºC, 5 s: 59ºC, 20 s)		80 bp
		CTGGAGGGCCACATCTTTTA	×40 cycles		

gadd45al	3B	CTCGGTGATTAAGGCTCTGG	95ºC, 30s:(94ºC, 5 s: 59ºC, 20 s) ×40 cycles		110 bp
		CGTTATCAGGGTCCACATTG			
gadd45b	3B	CCTCGTCACTAACTCTCAATGC	95ºC, 30s:(94ºC, 5 s: 59ºC, 20 s) ×40 cycles		134 bp
		CGCGAATAGTTCAGCGTTCT			
gadd45bl	3B	GCACTGAGAGAAAAATGGAGAC	95ºC, 30s:(94ºC, 5 s: 59ºC, 20 s) ×40 cycles		128 bp
		GCTGTCTGGATCCACATTCA			
gadd45g	3B	ACTACTGGCGATAGAATGCAG	95ºC, 30s:(94ºC, 5 s: 59ºC, 20 s) ×40 cycles		129 bp
		GCTGTCTGGGTCAACATTCA			
gadd45gl	3B	TGCTTATCACGAAACCTGCT	95ºC, 30s:(94ºC, 5 s: 59ºC, 20 s) ×40 cycles		116 bp
		GGGCAGAGTGATCTCAGGAA			
mbd4	4B,C,D S1(A)	AAGAGACGCTCTTCCATGA	95ºC, 10 m:(94ºC, 30 s: 55ºC, 30 s: 72ºC, 30 s)×40 cycles: 72ºC, 7 m		340 bp
		TTATGATCGTCTGGAGTGAC			
aid	5B	AGTGTGCTCATGACCCAGA	95ºC, 10 m:(94ºC, 30 s: 65ºC, 30 s: 72ºC, 30 s)×40 cycles: 72ºC, 7 m		200 bp
		CACAACGCACCCAAGTGA			
EF1a1	5D	TGCAGACTTTGTGACCTTGC	98ºC, 30 s:(98ºC, 5 s: 67ºC, 10 s: 72ºC, 1 m)×25 cycles: 72ºC, 7 m		1,439 bp
neurod2	6A,B S2	GTTTTTAAATAGGTATAGGT	95ºC, 10 m:(94ºC, 30 s: 54ºC, 30 s: 72ºC, 1 m)×40 cycles: 72ºC, 7 m	19	460 bp
		CAAACAAAATTACATACCTA			
		AGGTATAGGTTAGGTTATGT (nest)	95ºC, 10 m:(94ºC, 30 s: 53ºC, 30 s: 72ºC, 30 s)×35 cycles: 72ºC, 7 m		438 bp
		CATACCTACTCTTATACCAAA (nest)			
sox1a		TTTTGTTTAAAAAGGATAAGTAT	95ºC, 10 m:(94ºC, 30 s: 50ºC, 30 s: 72ºC, 1 m)×35 cycles: 72ºC, 7 m	43	555 bp
		AATACATACTAATCATCTCT			
		GTTAGAGGTTGGAGAGTTT (nest)	95ºC, 10 m:(94ºC, 30 s: 55ºC, 30 s: 72ºC, 30 s)×35 cycles: 72ºC, 7 m		459 bp
		TAATCATCTCTCTCAAATCTC (nest)			
atoh1a		AAAGATTGTTTGGGGTAAT	95ºC, 10 m:(94ºC, 30 s: 50ºC, 30 s: 72ºC, 1 m)×35 cycles: 72ºC, 7 m	13	405 bp
		AATTATTATACCCAAACTCTA			
mbd4	S1(A)	ACTGGTCTTGCGCTTCTCTACAGTTCTCTTCAG (5’RACE)	(94ºC, 5 s: 65ºC, 10 s: 72ºC, 3 m) ×28 cycles		~1.9 kb
		GCTGGAGAAGAGATGAGGCAGAAACACTC (3’RACE)	95ºC, 10 m:(94ºC, 30 s: 66ºC, 30 s: 72ºC, 1 m)×40 cycles: 72ºC, 7 m		~750 bp
mbd4	S1(B)	TGTTCTAACCCGCTTCTGCT	95ºC, 10 m:(94ºC, 30 s: 55ºC, 30 s: 72ºC, 30 s)×40 cycles: 72ºC, 7 m		multiple
		TTTCTCCGCGTCTCTTCTTC			

rpl13a	3A,B<br>5C	TCTGGAGGACTGTAAGAGGTATGC<br>AGACGCACAATCTTGAGAGCAG	95ºC, 30s:(94ºC, 5 s: 59ºC, 20 s) ×40 cycles		148 bp
